# Cardiovascular safety of testosterone therapy—Insights from the TRAVERSE trial and beyond: A position statement of the European Expert Panel for Testosterone Research

**DOI:** 10.1111/andr.70062

**Published:** 2025-05-15

**Authors:** Michael Zitzmann, Giulia Rastrelli, Robert D Murray, David Edwards, Yacov Reisman, Preethi Mohan Rao, Alexander Sahi, Thomas Hugh Jones, Alberto Ferlin, Eleni Armeni, Emiliano Corpas, Jann‐Frederik Cremers, Janine David, Stefan Arver, Leen Antonio, Giovanni Corona

**Affiliations:** ^1^ Centre for Reproductive Medicine and Andrology, University Hospital of Muenster Muenster Germany; ^2^ Department of Experimental and Clinical Biomedical Sciences University of Florence; ^3^ Andrology and Gender Endocrinology Unit, Careggi Hospital Florence Italy; ^4^ Department of Endocrinology Leeds Centre for Diabetes & Endocrinology, Leeds Teaching Hospitals NHS Trust Leeds UK; ^5^ Claridges Barn Chipping Norton, Oxfordshire UK; ^6^ Sexual Medicine, Flare‐Health Amsterdam Netherlands; ^7^ Center for Sexual Health, Reuth Rehabilitation Hospital Tel‐Aviv Israel; ^8^ Robert Hague Centre for Diabetes and Endocrinology, Barnsley Hospital NHS Trust Barnsley UK; ^9^ University of Sheffield Sheffield UK; ^10^ Urology and Andrology Cologne Germany; ^11^ Division of Clinical Medicine, School of Medicine and Population Health, University of Sheffield Sheffield UK; ^12^ Department of Medicine University of Padova Padova Italy; ^13^ Unit of Andrology and Reproductive Medicine, University Hospital of Padova Padova Italy; ^14^ Department of Endocrinology and Diabetes Royal Free Hospital London UK; ^15^ School of Health Sciences, College of Medicine and Health, University of Birmingham Birmingham UK; ^16^ Endocrinología del Hospital HLA de Guadalajara; ^17^ Endocrinología de la Facultad de Medicina de Alcalá Madrid España; ^18^ Porthcawl Medical Centre ,South Wales Porthcawl UK; ^19^ Princess of Wales Hospital, South Wales Bridgend UK; ^20^ Center for Andrology and Sexual Medicine at Karolinska University Hospital Stockholm Sweden; ^21^ Department of Chronic Diseases and Metabolism Clinical and Experimental Endocrinology, KU Leuven Leuven Belgium; ^22^ Department of Endocrinology University Hospitals Leuven Leuven Belgium; ^23^ Endocrinology Unit, AUSL Bologna Bologna Italy

**Keywords:** cardiosvascular risk, classical hypogonadism, functional hypogonadism, testosterone, testosterone therapy

## Abstract

**Introduction:**

Testosterone therapy has become a cornerstone treatment for men with hypogonadism, offering significant benefits such as improved sexual function, mood, muscle mass, and bone density. However, concerns about its cardiovascular safety have historically tempered its use. This position statement synthesizes the current evidence on the cardiovascular safety of testosterone therapy, drawing from key studies including the TRAVERSE trial, other trials, and recent meta‐analyses.

**Background and importance:**

Testosterone therapy aims to restore testosterone levels in men with hypogonadism, a condition associated with increased cardiovascular and metabolic risks. Early research produced mixed results, with some studies suggesting a potential increase in cardiovascular events such as myocardial infarction and stroke, while others indicated possible cardiovascular benefits, particularly in men with coexisting conditions like metabolic syndrome and type 2 diabetes.

**Findings from recent studies:**

The TRAVERSE trial, a large‐scale, randomized, placebo‐controlled study, provided robust evidence that testosterone therapy does not significantly increase the risk of major adverse cardiovascular events. Testosterone therapy was found to effectively mitigate anemia in hypogonadal men, highlighting a dual benefit of increasing red blood cell production while managing cardiovascular risks. The findings from the TRAVERSE trial align with those from previous meta‐analyses that concluded that testosterone therapy is safe and does not increase cardiovascular risk.

**Consensus and clinical implications:**

There is consensus that testosterone therapy, when prescribed to appropriately selected patients and monitored regularly, is safe from a cardiovascular standpoint, with the potential benefits outweighing the risks when the therapy is used responsibly. Current guidelines recommend individualized treatment plans with careful monitoring, especially of hematocrit levels. This position statement amalgamates previous knowledge with current data and is in agreement with recent United States Food and Drug Administration label changes for testosterone products.

## 1 INTRODUCTION

### 1.1 Background of testosterone therapy

Testosterone (T) is a natural hormone that is essential to maintain physical and emotional wellbeing in men, regardless of age. Male hypogonadism is an endocrine condition of testosterone deficiency with the potential to cause multiple morbidities and psychosocial complaints. The condition can be of primary (testicular), secondary (hypothalamic–pituitary) or so‐called functional origin (as result of inflammatory conditions, obesity, or chronic illness). Testosterone therapy (TTh) has been increasingly recognized as a pivotal intervention in the management of hypogonadism in men, a condition characterized by low levels of serum T accompanied by clinical symptoms such as sexual dysfunction, fatigue, mood disturbances, and low motivation/vitality, anemia, and osteopenia/osteoporosis. Hypogonadism can, hence, significantly impair quality of life^1^, ^2^, ^3^, ^4^ and has been associated with increased risk of metabolic and cardiovascular (CV) diseases (CVD) as well as increased mortality.^5^

Available guidelines emphasize the need of diagnosing and managing hypogonadism with a clear understanding of the risks and benefits associated with TTh.^1^, ^2^, ^3^ TTh should not be initiated in case of desired paternity, unclear processes of the prostate, mammary gland, or high hematocrit (Hct) level. TTh is proven to be effective to ameliorate the above named complaints when performed according to these guidelines. In organic primary or secondary hypogonadism, TTh has not been challenged. In functional hypogonadism, which is most often, but not exclusively, found in older men, treatment of the underlying condition/comorbidity has been suggested to be mandatory prior to starting a TTh.^1^ As these men might have, because of age and the underlying comorbidity, an increased CV risk, it is essential to elucidate to whether TTh might affect this CV risk.

TTh aims to restore T levels to the physiological range, thereby alleviating the symptoms of hypogonadism. The clinical application of TTh has been extensively studied also in functional hypogonadism, revealing its benefits in improving bone density, muscle mass, sexual function, and mood.^6^ However, the therapy is not without controversy, particularly regarding long‐term safety. Concerns about potential adverse effects, such as CV events and prostate health, have fueled ongoing debates and research efforts.^6^, ^7^

CV safety, in particular, still represents the most challenging topic due the lack of long‐term specific trials, with CV safety as the primary endpoint, and the different positions raised by the the United States Food and Drug Administration (US FDA) and the European Medicine Agency (EMA). In 2013, the US FDA Drug Safety Communication raised concerns about using T products for low T due to aging and requireda label change to include information of possible increased risk of heart attack and stroke.^8^ On the other hand, the EMA's pharmacovigilance risk assessment committee reviewed the same evidence and concluded there was inconsistent evidence that TTh increases CV risk.^9^

This statement aims to clarify the current knowledge of TTh regarding CV safety, including the evidence gained from the Testosterone Replacement Therapy on the Incidence of Major Adverse CV Events (MACE) and Efficacy Measures in Hypogonadal Men trial (TRAVERSE), the largest placebo‐controlled, randomized, study to date.^7^

The TRAVERSE study is groundbreaking but limited in scope. While it provides robust evidence regarding CV safety, it does not offer actionable guidance on:

1. Monitoring protocols: timing for testosterone levels and Hct checks.
2. Practical considerations for at‐risk populations: managing older patients, those with functional hypogonadism, or with significant comorbidities.
3. Contextualizing prior conflicting evidence: synthesizing findings from earlier studies to resolve historical controversies.
4. Our panel provides clear, actionable recommendations for clinical practice based on TRAVERSE, aligned with existing guidelines simultaneously streamlined for real‐world application.

Since the publication of the TRAVERSE Trial, the only guideline that was updated was that of the EAU 2024. Despite the latter point of the present paper represents a concise expert statement addressing the above mentioned points.

### 1.2 Importance of CV safety in TTh

The CV safety of TTh is a critical issue in managing hypogonadism in men, as T levels decline with age or illness and comorbidities. Deciding to initiate TTh requires weighing the symptomatic benefits against potential risks. Continued research on the long‐term CV impacts of TTh, especially for patients with existing CV conditions, remains essential. Historically, T's role in CV health has been contentious, with early studies yielding mixed results on its impact also in guidelines (e.g., Ref. 1). In particular, retrospective studies hinted at links between low T serum concentrations and increased risks of events such as myocardial infarction and stroke, prompting caution among regulatory bodies and clinicians. In particular spurred by flawed or insufficient studies between 2010 and 2014,^10^, ^11^, ^12^ (see below, Section 2) suggesting TTh's negative CV effects, a precautionary stance was taken by the US FDA.^8^ Another important limitation of the study by Basaria et al.^10^ is the enrollment of very old, institutionalized patients in bad health conditions (the presence of physical limitation was an inclusion criteria) and with a high risk profile for CV events at baseline independently from the T therapy.

Such results led to intensified research efforts, with the US FDA requesting prospective studies of sufficient size and duration to evaluate whether T products are associated with an elevated risk of CV events. Hence, the TRAVERSE study was thereafter planned and organized.^13^

## 2 REVIEW OF PREVIOUS RESEARCH ON TTh AND CV RISK

Before the TRAVERSE trial, the link between TTh and CV risk was widely debated due to mixed findings. Key publications influenced this understanding: Wang et al.^14^ conducted a comprehensive review on TTh's effects on CV health in hypogonadal men, particularly those with type 2 diabetes mellitus (T2DM). The review highlighted associations between low T and higher CV risk, emphasizing TTh's potential benefits, such as reduced visceral fat, improved insulin sensitivity, and better lipid profiles, suggesting CV improvements in this population. Zitzmann^15^ focused on the relationship between T deficiency, metabolic syndrome (MetS), and CV risk, underscoring that TTh might improve insulin sensitivity, reduce body fat, and enhance lipid profiles. This review supported TTh as a treatment for hypogonadism and as a strategy for mitigating CV risk in men with MetS.

Two retrospective studies of prescription databases showed a higher risk of CV events in men receiving TTh, with the risk increasing early after the treatment was started.^11^, ^12^ However, in the paper by Vigen et al.^11^, it appeared that the initial dataset included women and more than 1000 men were excluded erroneously, leading to the publication of corrections. Furthermore, the actual adverse event rate was only half as great in the T group compared with the untreated group, but the opposite conclusion was drawn based on statistical modelling that reversed the direction of the association. The dataset used in the study by Finkle et al only included data on prescriptions, not on actual treatment, nor did it contain information about hypogonadal symptoms or CV risk factors. The study by Basaria et al.^10^ included doses of T that were higher than the label dose, CV events were not the primary study target and were recorded in a much less than optimal manner.

The T‐Trials by Snyder et al.^6^ assessed TTh's effects on sexual, physical, and cognitive function in older men, monitoring CV events as secondary outcomes (myocardial infarctions, stroke). EAA guidelines^2^ also suggested that TTh could offer CV benefits, especially for men with MetS or T2DM, by improving lipid profiles, insulin sensitivity, and body composition.

One of the most recent and comprehensive analyses on the CV safety of TTh was conducted by Corona et al.^16^ This updated systematic review and meta‐analysis included 106 randomized, placebo‐controlled trials, encompassing over 8000 men treated with TTh and more than 7000 on placebo. The study specifically examined the incidence of major adverse CV events (MACE) and non‐fatal arrhythmias, including atrial fibrillation (AF). The meta‐analysis found no significant difference in the occurrence of MACE between the TTh and placebo groups, reinforcing the notion that TTh does not increase overall CV risk. Despite this, when the data from all trials were pooled, the increased risk was not statistically significant for arrhythmias and AF (MH‐OR 1.61[0.84;3.08] and 1.44[0.46;4.46]) respectively. Additionally, no correlation between endogenous T levels and AF incidence was observed after adjusting for confounding factors. These findings further support the safety of TTh in terms of CV risk.

## 3 A BROADER SAFETY CONSIDERATION IN TTh: Hct LEVELS AND THROMBOEMBOLIC EVENTS

TTh is known to elevate Hct levels by stimulating erythropoiesis, which, while beneficial in treating anemia in men with hypogonadism, also raises concerns about hyperviscosity and thromboembolic risk, such as deep vein thrombosis and pulmonary embolism. Findings from the TRAVERSE trial^17^ confirmed that while TTh modestly increased Hct, this did not lead to a significant rise in thromboembolic events among participants.

Kohn et al.,^18^ however, retrospectively analyzed data from a claims database and observed that Hct rises in men undergoing TTh were linked to an increased risk of MACE, including myocardial infarction, stroke, or death, particularly when Hct increased significantly within the first 24 months. The HEAT (HEmatopoietic Affection by Testosterone) Registry^19^ also examined TTh's effects, finding both transdermal and intramuscular formulations raised Hct, though thromboembolic events remained low with appropriate monitoring.

In the Australian T4DM study, during which more than 1000 men received intramuscular injections of T undecanoate versus placebo, 22% using T had an elevated Hct > 0.52. These blood samples were taken under fasting conditions in a hot country. On repeat samples in a non‐fasting condition the incidence of an elevated Hct fell to 2.5%^20^.

In addition, a meta‐analysis scrutinizing data derived from placebo controlled RCTs previously showed that when the analysis was limited to the studies enrolling only hypogonadal (T < 12 nmol/L) patients at baseline treated with transdermal T preparations, the risk of elevated Hct (>52%) was not found (hazard ratio [HR] = 4.89 [95% CI = 0.83–28.91]; *p* = 0.08]).^21^

This evolving research emphasizes the importance of closely monitoring Hct in men on TTh. While trials like TRAVERSE generally support TTh's CV safety, Kohn et al.’s findings stress that even modest Hct (>52%) increases can elevate CV risk if left unmonitored. For clinicians, this underscores the need for vigilant Hct management, especially in the first year of therapy, potentially involving TTh dose adjustments or temporary discontinuation to maintain safe Hct levels and mitigate MACE risk.

## 4 OVERVIEW OF THE TRAVERSE TRIAL AND ITS SIGNIFICANCE

The TRAVERSE trial stands as a landmark study in TTh, particularly concerning CV safety. It employed a large‐scale, randomized, double‐blind, placebo‐controlled approach.^7^ Enrolling men with clinically confirmed hypogonadism, the trial aimed to assess the incidence of MACE—including myocardial infarction, stroke, and CV death—over an extended follow‐up. Unlike prior studies, where CV safety was always a secondary outcome, the TRAVERSE trial's long‐term design enabled a robust assessment of TTh's CV effects.

### 4.1 Study design and population

TRAVERSE enrolled 5246, 45–80‐year‐old men (mean age 63.3 years; 47% older than 65 years of age) with functional hypogonadism as above mentioned. Most suffered from existing CV disease or they were at high risk of CV events. Mean body mass index was 35 kg m^−2^. Approximately 70% had T2DM (HbA1c 6.5–11%), and 90% had hypertension and dyslipidemia. Participants received either placebo or T gel (a 1.62% gel), titrated to maintain physiological serum T levels (350–750 ng/dL) for a mean of 22 months. Men with recent CV events, uncontrolled hypertension, prostate cancer, or elevated PSA levels were excluded.

### 4.2 Key results related to CV outcomes

The TRAVERSE trial offered critical insights into the CV safety of TTh for men with hypogonadism. The primary end point was based on the first occurrence of a composite of death from CV causes, nonfatal myocardial infarction, or nonfatal stroke, assessed in a time‐to‐event analysis. A secondary CV end point was the first occurrence of any component of the composite of primary end point and coronary revascularization. Results showed no statistically significant increase in CV risk in the TTh group compared with placebo when either primary or secondary end points were considered.^7^

Other secondary outcomes included the risk related to myocardial infarction, stroke, CV death, heart failure, and non‐fatal arrhythmias. No significant differences were seen in CV death or heart failure. While there was a minor, but significant, increase in AF and non‐fatal arrhythmias in the active harm, lipid profiles improved modestly with increased high density lipoprotein levels, and blood pressure and well as glycometabolic control remained stable across groups.

The TRAVERSE trial took place during the COVID pandemic. New research shows that the occurrence of COVID‐19 was associated with substantially increased risk of MACE, venous thromboembolism, AF, and acute kidney injury in the total TRAVERSE cohort; the HRs for the risk of venous thromboembolism and acute kidney injury were particularly high in infected subjects while the relation of such events to TTh was no longer visible.^22^

Overall, the TRAVERSE trial provides robust evidence that TTh, when correctly administered, does not increase MACE risk, challenging previous concerns and affirming TTh's safe use in hypogonadism management. This evidence supports prescribing TTh with increased confidence in CV safety, provided patients are carefully selected and monitored.

Notwithstanding, some limitations of the TRAVERSE trial have to be mentioned:

1. TRAVERSE included an older, high‐risk population (mean age 63.3 years, BMI 35, ∼70% with T2DM), limiting generalizability to younger, healthier hypogonadal men. In these men, CV events may seem less likely, but it is also possible that monitoring is less strict
2. Exclusion of men with low baseline CV risk makes the findings less applicable to this subgroup.
3. The study does not address long‐term CV outcomes beyond ∼22 months.
4. Endpoints like arrhythmias and heart failure were underpowered for definitive conclusions.

## 5 TREATMENT OF HYPOGONADAL MEN WITH ANGINA AND CHRONIC HEART FAILURE

Several randomized placebo‐controlled trials (RCTs) of TTh have been conducted in men with CVD.^23^ If T had any adverse clinical effects on heart or the vascular tree then these would be the study cohorts that would most likely identify problems. The published RCT's range in duration mainly between 3 and 24 months but most are mainly in the 3–12 month bracket.

There is clear evidence that TTh with treatment periods from 1 to 12 months improves exercise induce cardiac ischemia, measured by exercise treadmill testing.^24^, ^25^, ^26^, ^27^ Although angina frequency was not affected there was a significant improvement in quality of life as assessed by SF‐36 in all domains.^28^ Specifically there were marked improvements in the pain perception and physical role‐limitation domain. In these studies there was no of any CV safety concerns with the few reported events between treatment and placebo groups having no significant differences.

A prolonged QTc that is a standardized measure of the variability in the QT‐interval is prolonged in men with hypogonadism.^29^ A prolonged QTc is associated with an increased risk of ventricular tachycardia, ventricular fibrillation, and Torsades du Points, which can each cause sudden death. TTh two RCTs of men with chronic angina and/or cardiac failure reduced QTc and shortened QT and QT intervals.^30^, ^31^

RCTs of TTh in men with chronic heart failure of between 1 and 12 months duration with have not reported any safety concerns.^25^, ^32^, ^33^, ^34^, ^35^, ^36^ The study numbers have been relatively small between 20 and 76 with the longest 12 month trial having the largest study number of 76. All but one study have reported significant benefits, which include improved functional exercise capacity, New York Heart Class of heart failure, Vo2max, left ventricular length, insulin resistance, and maintenance of blood pressure compared with placebo.^23^, ^37^ An RCT of acute administration of TTh versus placebo in men with chronic heart failure reduces peripheral vascular resistance and improves cardiac output.^38^ None of these RCT's reported any increase in CV events in those men treated with TTh.

T has been demonstrated to have acute and chronic actions as an arterial vasodilator increasing coronary blood flow.^39^ T has been shown to have calcium channel blocking and potassium channel opening effects, which lead to arterial vasodilation.^39^, ^40^ Specifically T has been shown to act as a L‐calcium channel blocker by inhibiting the nifedipine binding site.^41^, ^42^ ^;^ ^43^ T also has several other direct actions on the CV system.^23^

## 6 EXPERT PANEL DISCUSSION

In September 2024, an international European Group of Experts in the Field of TTh (European Expert Panel for Testosterone Research [PaTeR]) convened in London to discuss and draft this position statement on the TRAVERSE study and its impact.

### 6.1 Discussion and interpretation of the TRAVERSE findings in the context of existing literature

The TRAVERSE trial represents a major advancement in assessing the safety of TTh, particularly in relation to CV risk and Hct levels. It builds on previous research by providing a large‐scale, randomized, long‐term analysis that offers a more nuanced understanding of TTh's risks and benefits. Consistent with other studies, including meta‐analyses and guidelines (e.g. Refs. 3, 6, 16), TRAVERSE found no significant increase in MACE associated with TTh. This result supports previous findings that, with appropriate administration, TTh does not elevate CV risk.

The trial also clarified concerns over Hct increases, a common effect of TTh, finding that modest Hct elevations did not correlate with higher thromboembolic events, a conclusion echoed by the HEAT Registry.^19^ This underscores the importance of Hct monitoring to maintain safety.

TRAVERSE's findings are likely to influence future clinical guidelines by supporting a balanced approach to TTh, where its benefits for men with symptomatic hypogonadism—such as reduced fatigue, improved libido, and muscle strength—can be considered with greater confidence. This aligns with the European Academy of Andrology and European Association of Urology guidelines as well as the SIAMS guideline,^2^ EAU 2024,^3^ which recommend TTh when underlying health conditions are managed appropriately.

Recent reviews, such as by De Silva et al.,^44^ also highlight that TTh can improve sexual function, bone density, and insulin sensitivity in men with functional hypogonadism, though they emphasize the need for holistic management, including lifestyle changes.

The aim of this consensus is thus to discuss and create a position statement on the TRAVERSE study in the light of other trials. Hence, many statements of this position stated are based on studies different from the TRAVERSE. While the TRAVERSE study serves as the cornerstone of our consensus, its findings must be contextualized with other key studies and pivotal guidelines (e.g., Refs. 3, 6, 16, 19) to provide a comprehensive view.

We highlight nuances not addressed by TRAVERSE, such as monitoring strategies for Hct and PSA levels and support findings (e.g., no significant thromboembolic risk) with independent datasets.

Based on recent findings from the TRAVERSE trials, the US FDA is implementing several significant changes to testosterone product labeling:

- Addition of TRAVERSE trial results to all testosterone product labels
- Retention of “limitation of use” language for functional hypogonadism
- Removal of language from the Boxed Warning related to increased risk of adverse CV outcomes^45^

### 6.2 Recommendations for clinicians

With evidence supporting the CV safety of TTh, notably from the TRAVERSE trial, clinicians can manage hypogonadism with greater confidence.

This position statement, albeit not written as a guideline, provides new aspects regarding recommendations:

We provide novel contributions compared with guidelines: Practical suggestions for testosterone monitoring (e.g., timing relative to gel or injectable formulations). Thresholds for managing Hct (≤54%) and recommendations for addressing erythrocytosis (e.g., dose adjustments, phlebotomy). Distinction between functional and organic hypogonadism for individualized treatment.

1. Patient selection and assessment
  - *Accurate diagnosis*: Confirm hypogonadism with consistent symptoms (e.g., fatigue, low libido) and low serum testosterone according to guidelines. The EAU and EAA guidelines mention 12.0 nmol/L as cut‐off.^2^, ^3^, ^4^
  - *A distinction* between functional and organic hypogonadism for individualized treatment is required.^46^ The latter forms can be primary or secondary hypogonadism. This “classical form” of hypogonadism can be of testicular origin or due to hypothalamic–pituitary failure, thus have definitive etiologies. Functional hypogonadism represents a clinical condition associated with comorbidities (e.g., obesity, MetS), which can act either at central or peripheral level, potentially requiring lifestyle intervention prior to TTh.
  - *Severe hyperprolactinemia* can cause secondary hypogonadism . It can be treated by adequately addressing the source (mostly adenomas of the pituitary) or the production (using cabergolin or related drugs). In many cases, a TTh is not required then.
  - *Lifestyle modifications*: We prioritize lifestyle modifications as an initial intervention for functional hypogonadism, emphasizing weight management and physical activity.
  - *Comprehensive evaluation*: Assess overall health, focusing on CV risk factors like hypertension, DM, sleep apnea and history of CV disease.
  - *Exclude contraindications*: Avoid TTh in men with untreated sleep apnea, severe heart failure, a recent CV event, or hormone‐sensitive cancers unless under specialist guidance.
2. Individualized treatment planning
  - *Tailored formulation*: Select the most suitable formulation and dose based on patient needs, preferring transdermal, or long term injectable options.
  - *Dosing*: Transdermal Gel preparations can be easily titrated in daily dose, injectable forms of treatment should be titrated by modulating injection intervals
  - *Shared decision‐making*: Discuss TTh's benefits, risks, and the need for ongoing monitoring, ensuring patient involvement in treatment decisions.
3. Monitoring and follow‐up
  - *Regular monitoring*: Schedule follow‐ups for testosterone, Hct, lipid profile, and PSA levels. Check Hct every 3–6 months in the first year, then annually, keeping levels below 54% to minimize thromboembolic risk. Adjust TTh dosing based on these measurements.
  - *Measuring testosterone levels in serum*:
    ✓ Gel formulations: Measure trough levels ∼2–4 h after the last application.
    ✓ Injectables: Timing depends on the formulation. For long‐acting T undecanoate, measure preferably 1 week before the next injection (the next injection might be adjourned in case of levels still being within the higher normal range). For short acting injections, measure directly before the next injection.
  - *CV monitoring*: Regularly assess CV health, especially in patients with pre‐existing conditions, monitoring blood pressure and glucose and lipid levels.
4. Managing side effects and complications
  - *Erythrocytosis*: If Hct exceeds safe limits, adjust TTh dosage, consider a different formulation, or temporarily stop therapy. Phlebotomy may be warranted to reduce Hct.
  - *Prostate health surveillance*: Monitor PSA levels and perform prostate exams. PSA levels are likely to increase with TTh. PSA increases ≤1.4 ng/mL/year are generally considered safe. Increases beyond this threshold warrant urologic evaluation. Lower urinary tract symptoms (LUTS) require regular monitoring.
5. Long‐term considerations
  - *Reevaluation*: Periodically review TTh necessity, especially in older patients or those with new health issues, and discuss tapering if risks outweigh benefits.
  - *Lifestyle modifications*: Encourage lifestyle changes, such as weight management and regular physical activity. Especially in functional hypogonadism, where the underlying morbidity is most often obesity‐associated, this is a paramount key to success of treatment and must precede TTh.
6. Staying informed
  - *Continuous education*: Stay updated on evolving research and guidelines in TTh to align practice with the latest evidence.

*Conclusion*: By implementing these suggestions, clinicians can manage hypogonadism effectively, optimizing TTh's benefits while minimizing risks. Evidence from the TRAVERSE trial supports the CV safety of TTh when appropriately prescribed and monitored, reinforcing the need for individualized care, regular monitoring, and patient education for optimal outcomes.

However, careful prescribing and monitoring remain essential to balance benefits and risks effectively.

## 7 FUTURE RESEARCH DIRECTIONS

While substantial progress has been made in understanding the CV safety of TTh, key areas for future research remain to further refine its application and optimize patient outcomes.

1. *Long‐term CV outcomes*: The TRAVERSE trial and similar studies have reassured us of TTh's short‐to‐medium‐term CV safety, yet long‐term effects, especially across diverse populations, require further investigation. Extended follow‐up studies are needed to assess the potential for long‐term CV safety in men undergoing decades‐long TTh.
2. *Personalized medicine and genetic factors*: Genetic factors, like the CAG repeat polymorphism in the androgen receptor (AR) gene, which affects receptor sensitivity to T, merit deeper study. Future research should explore how genetic polymorphisms influence TTh's efficacy and safety, aiming for a personalized approach that considers not only clinical symptoms and T levels but also genetic profiles.^15^, ^47^, ^48^
3. *Hct and thromboembolic risks*: Although current evidence indicates that Hct increases with TTh can be managed safely, refining thresholds for Hct monitoring and intervention remains a priority. Future studies should aim to develop guidelines tailored to patients at higher thromboembolic risk.
4. *Broader health implications of TTh*: Expanding research to assess TTh's impact on cognitive function, bone health, and mortality will deepen understanding of its overall health effects. Such insights would allow for a more comprehensive balancing of TTh's risks and benefits for varied patient populations.
5. *Comparative effectiveness of TTh formulations*: Comparative studies on different TTh formulations (e.g., gels, injections, patches) are needed to identify the safest and most effective options for patients with specific comorbidities. This can guide formulation choice in tailored clinical contexts.

By focusing on these research areas, the TTh evidence base can be enhanced, leading to safer, more effective treatment approaches for men with hypogonadism. Integrating genetic factors, broader health outcomes, and real‐world data from registries will also support optimized, long‐term TTh use, particularly as RCT trials become increasingly challenging due to ethical and financial considerations.

## 8 COMPARISON WITH THE STATEMENT OF THE ANDROGEN SOCIETY

An another comprehensive position statement on the CV safety of TTh exists: it was issued by the US‐based Androgen Society (AS).^49^ It is essential to compare this paper with ours as both manuscripts reflect a paradigm shift following the publication of the TRAVERSE trial, emphasizing that TTh is not associated with an increased risk of major adverse cardiovascular events (MACE) in hypogonadal men. However, while they align on the principal findings, they differ in tone, scope, emphasis, and intended clinical application.

The Androgen Society adopts a declarative tone, framing the TRAVERSE findings as a definitive resolution of a decade‐long controversy. It critiques earlier studies that suggested harm and calls for regulatory reversal of US FDA warnings. The paper serves as a strong defense of TTh's safety, presenting exhaustive data and rather dismissing the need for further large‐scale CV trials.

In contrast, the PaTeR panel presents a more cautious and clinically integrative stance. It acknowledges the strength of TRAVERSE but also its limitations, such as the older, high‐risk study population and relatively short follow‐up. Unlike the AS statement, the PaTeR urges individualized care, especially distinguishing between organic and functional hypogonadism, recommending lifestyle interventions prior to therapy in the latter group. It also provides practical clinical guidance—on monitoring testosterone levels, Hct, PSA, and managing erythrocytosis—making the document highly applicable in daily practice. It calls for further research, particularly on long‐term outcomes, genetic polymorphisms influencing androgen sensitivity, and formulation‐specific safety profiles.

Both documents agree that flawed earlier studies fueled undue criticism of TTh and affirm that low testosterone is associated with poor CV outcomes. They also address concerns about AF and thromboembolic events during TTh, though PaTeR explores these in more detail with a more cautious stance, also regarding the impact of COVID‐19 on TRAVERSE results.

In summary, both position papers provide robust, evidence‐based support for the CV safety of TTh. The Androgen Society's statement is a forceful rebuttal of past concerns, positioned as a scientific and regulatory manifesto. The PaTeR panel's document is more of a clinical roadmap, embedding the TRAVERSE results within a broader, ongoing conversation about the nuanced and individualized use of testosterone in practice. Thus, while the AS paper aims to reshape perception and policy, the PaTeR statement focuses on clinical implementation. Together, they mark a significant shift toward accepting TTh as safe and beneficial when prescribed judiciously and monitored appropriately.

## 9 FINAL POSITION OF THE CONSORTIUM ON TTh AND CV SAFETY

Recent evidence has provided more nuanced insights into the CV safety of TTh. This landmark study and others like it have contributed to a better understanding of the conditions under which TTh can be safely administered.

The expert panel concludes that TTh is safe from a CV standpoint when prescribed to men with clinically confirmed hypogonadism and managed appropriately. The findings from the TRAVERSE trial and supporting studies provide strong evidence that TTh does not significantly increase the risk of MACE.

The expert panel emphasizes the importance of individualized treatment, regular monitoring of CV and hematological parameters, and careful patient selection to ensure the safe and effective use of TTh. In light of the current evidence, TTh should be considered a viable and beneficial treatment option for men with hypogonadism, provided that these precautions are followed.

## AUTHOR CONTRIBUTIONS

All PaTeR members contributed to reviewing literature, drafting, and writing this position statement.

## Data Availability

Data sharing not applicable to this article as no datasets were generated or analyzed during the current study.
